# The effect of metronomic *versus* standard chemotherapy on the regulatory to effector T-cell equilibrium in cancer patients

**DOI:** 10.1186/2162-3619-3-3

**Published:** 2014-01-23

**Authors:** Anna Koumarianou, Maria-Ioanna Christodoulou, Pavlos Patapis, Iordanis Papadopoulos, Elissavet Liakata, Athina Giagini, Anastasia Stavropoulou, Nikiforita Poulakaki, Nikolaos Tountas, Nikolaos Xiros, Theophanis Economopoulos, Dimitris Pectasides, Ourania E Tsitsilonis, Vassiliki Pappa

**Affiliations:** 1 Fourth Department of Internal Medicine, Attikon University Hospital, Rimini 1 Street, 12462 Athens, Greece; 2 Medical Oncology Unit, Second Department of Internal Medicine Propaedeutic and Research Institute, Attikon University Hospital, Rimini 1, 12462 Athens, Greece; 3 Third Department of Surgery, Attikon University Hospital, Rimini 1, 12462 Athens, Greece; 4 Fourth Department of Surgery, Attikon University Hospital, Rimini 1, 12462 Athens, Greece; 5 Department of Microbiology, Medical School, Athens University, 75 Mikras Asias Street, 11527 Athens, Greece; 6 Third Department of Obstetrics and Gynecology, Attikon University Hospital, Rimini 1, 12462 Athens, Greece; 7 Athens Medical Centre, 5-7 Distomou Street, 15125 Athens, Greece; 8 Second Department of Internal Medicine, Athens University, ‘Hippokration’ General Hospital 114, Vassilissis Sophias Avenue, 115 27 Athens, Greece; 9 Department of Animal & Human Physiology, Faculty of Biology, University of Athens, Panepistimiopolis, 15784 Athens, Greece; 10 Hematology Unit, Second Department of Internal Medicine Propaedeutic and Research Institute, Attikon University Hospital, Rimini 1, 12462 Athens, Greece

**Keywords:** Metronomic and standard chemotherapy, Regulatory CD4^+^CD25^+^ T-cells, Effector CD4^+^CD25^-^ T-cells, Regulatory T-cell suppression, Anti-tumour immunity

## Abstract

**Background:**

The host’s immune system is crucially involved in cancer development and progression. The ratio of regulatory to effector T-cells, as well as the interplay of T-cells with therapeutic agents, impact on cancer prognosis. The current study aimed to comparatively investigate the effect of metronomic and standard chemotherapy on the number and functionality of peripheral regulatory and effector T-cells in cancer patients.

**Methods:**

CD4^+^CD25^+^ regulatory and CD4^+^CD25^-^ effector T-cells were purified from the peripheral blood of 36 cancer patients and co-cultured in the presence of a polyclonal stimulus. The proliferative capacity and frequency of CD4^+^CD25^+^/CD4^+^CD25^-^ T-cells were analysed before and during various chemotherapeutic regimes, by ELISA and flow cytometry, respectively.

**Results:**

Chemotherapy shifted immune responses in favour of regulatory T-cells. The relative ratio of regulatory to effector T-cells increased, and the T-cell-mediated suppressive activity of regulatory on effector T-cells was augmented. This effect was more profound in metronomic than in standard chemotherapeutic approaches. Moreover, an association between the chemotherapy strategy followed and the mode of action of specific drugs (anti-mitotic, anti-DNA) was revealed.

**Conclusions:**

In comparison to standard chemotherapeutic strategies, metronomic approaches, though more patient-friendly, result in a significantly more prominent expansion of regulatory T-cells that aggravate the regulatory to effector T-cell imbalance. Our findings impact on the modulation of chemotherapy-treated patients’ anti-tumor immunity and, thus, may be proven useful for selecting the most advantageous drug-delivery strategy, particularly when immunotherapeutics are eventually to be applied.

## Introduction

Recently, immune evasion by malignant cells has been identified as one of the crucial hallmarks of cancer development [1]. This evasion may be mediated by immunoediting (i.e. the selection of non-immunogenic tumour-cell variants) or active immunosuppression of the immune response [2]. The CD4^+^CD25^+^ regulatory T-cells (Tregs) are the most extensively studied suppressive cells, required for the regulation of essential immune processes in allergies, infections, transplantations, autoimmune diseases and neoplasia [3]. Tregs control the activity of effector T-cells (Teffs) and other immune cells primarily through cell-to-cell contact, as well as by producing suppressive cytokines (e.g. interleukin-10; IL-10 and transforming growth factor-β; TGF-β) [4].

Increasing evidence suggests that cancer progression correlates with an increase in Treg activity and a decrease in Teff functions [5,6]. The percentage of Tregs is elevated in tumour tissues and/or in the peripheral blood of a variety of cancer entities and is associated with poor prognosis and marginal, if any, clinical response to adoptive immunotherapy [7,8]. Additionally, the ratio of CD8^+^ cytotoxic T-cells to Tregs is considered a predictor of the patients’ survival [7,9].

Currently, many anti-cancer therapeutic approaches are applied in the clinical setting, including immunotherapies, as anti-tumor vaccination, that require a functional immune system to generate objective responses [10-12]. However, conventional chemotherapy remains the standard-of-care and predominantly aims to block tumour-cell proliferation by targeting the spindle microtubules during cell mitosis, or by interfering in the DNA sequences during DNA replication in the S phase of the cell cycle. Recent data suggest that chemotherapy halts tumour progression also by inducing immune-mediated anti-cancer responses, such as immunogenic cancer-cell death and increased immune-susceptibility of tumour cells [13,14]. However, chemotherapy has also been associated with poor prognosis due to the selection of chemotherapy-resistant cancer cells, the impairment of Teffs and the activation of immune-suppressive mechanisms [12,13,15]. Along these lines, the effect of chemotherapy on Tregs, in terms of their frequency in the periphery and/or at the tumour site, is yet not fully defined and previous reports have generated conflicting results [16]. This may be a consequence of (a) the non-uniformity of the applied experimental approaches, including the methods used for Treg identification and/or the estimation of their rate; (b) the fact that these studies were confined to specific chemotherapy regimens; and (c) the negligence of the underlying functional state of the host’s immune system, which is vital for the fate of cancer patients treated with these regimens. Moreover, the functional consequences resulting from alterations in the Treg to Teff equilibrium, especially within the CD4^+^ T-cell compartment, have not been reported. The importance of such studies emerges also in view of novel means of chemotherapy administration. Indeed, as for today, chemotherapy is not only provided following standard intravenous cycles, but, alternatively, *via* a metronomic pattern in which the drug is chronically administered at relatively low, minimally toxic doses with no prolonged drug-free breaks. In an attempt to determine to which side the peripheral blood CD4^+^ Treg-Teff equilibrium tilts during anti-cancer therapies, we investigated the effects of metronomic (oral) *vs*. standard (intravenous) chemotherapy administration on the cell ratio of peripheral CD4^+^CD25^+^ Tregs and CD4^+^CD25^-^ Teffs, and on the suppressive capacity of peripheral Tregs over Teffs, in patients with solid tumours.

## Patients and methods

### Patients and blood samples

The study was approved by the Attikon University Hospital Ethics Committee and conducted in conformance with the Declaration of Helsinki Protocols. All samples were collected following informed consent. The enrolment criteria included newly diagnosed cancer patients with various tumours (n = 36) receiving either adjuvant or first-line chemotherapy; of these, 19 patients received metronomic treatment and 17 were treated with standard chemotherapy. Exclusion criteria included previous treatments with chemotherapy, steroids or other immunosuppressive agents. For the standard and metronomic chemotherapy arms, peripheral blood samples were collected from patients after the first cycle of chemotherapy and prior to administration of the second cycle. The treatment cycles applied for each chemotherapeutic agent are presented in Table 1. Control samples were obtained from patients just prior to treatment initiation, as well as from 13 healthy blood donors. Patient characteristics and treatment details are presented in Table 2.

**Table 1:** Chemotherapy treatment and drug classes

**Drug**	**Category**	**Frequency**	**Dose**	**Cancer type**
***Standard administration***				
Epirubicin	Anthracycline	2 W	100 mg/m^2^	Breast
Paclitaxel	Taxane	3 W	175 mg/m^2^	Breast
Carboplatin	Alkylating agent	3 W	AUC6	Lung, ovarian
***Metronomic administration***				
Vinorelbine	Vinca alkaloid	5D	30 mg	Breast, lung, prostate
Capecitabine	Anti-metabolite	14Q21	2000 mg	Colorectal
Temozolomide	Alkylating agent	OD	100 mg	Colorectal

2 W: every 2 weeks, 3 W: every 3 weeks, 5D: every 5 days, 14Q21: 14 days every 21 days, OD: once daily (the cycle of chemotherapy treatment was 3 weeks), AUC: area under the curve.

**Table 2:** Characteristics of patients included in the study

**Features**		**Type of cancer**				
	**Total**	**Breast**	**Lung**	**Colorectal**	**Ovarian**	**Prostate**
	**(n = 36)**	**(n = 16)**	**(n = 7)**	**(n = 6)**	**(n = 4)**	**(n = 3)**
***General***						
Age at diagnosis, median (range) in years	60 (46–86)	52 (46–66)	65 (55–74)	60 (50–86)	67 (59–79)	75 (69–75)
Gender, *n* (%)						
Male	14 (39)	0	6 (86)	4 (66)	0	3 (100)
Female	23 (61)	16 (100)	1 (14)	2 (33)	4 (100)	0
Early/advanced disease, *n* (%)						
Early	17 (47)	10 (63)	2 (29)	2 (33)	3 (75)	0
Advanced	19 (53)	6 (38)	5 (71)	4 (66)	1 (25)	3 (100)
Survival, *n* (%)						
Alive	29 (81)	15 (94)	7 (100)	4 (66)	1 (25)	2 (67)
Deceased	8 (19)	1 (8)	0	2 (33)	3 (75)	1 (33)
***Clinical status***						
PS^a^, median (range)						
At diagnosis/prior to treatment	1 (0–3)	0 (0–2)	1 (0–3)	1 (0–3)	0 (0–1)	2 (1–3)
During treatment	1 (0–3)	0 (0–2)	1 (0–2)	0 (0–3)	0 (0–1)	1 (1–1)
***Blood counts***						
WBC^b^ (×10^3^/μL), median (range)	7.1 (4.0–24.9)	7.1 (5.0–12.8)	8.2 (5.2–24.9)	5.8 (4.5–6.4)	7.8 (6.1–9.5)	7.2 (4.0–8.0)
Lymphocytes (×10^3^/μL), median (range)	1.5 (0.0–3.6)	1.4 (0.0–2.6)	2.1 (1.0–3.1)	1.2 (0.9–1.4)	1.8 (0.5–2.4)	1.5 (1.2–1.9)
% Lymphocytes, mean ± SE^c^	20.8 ±1.6	20.7 ± 3.6	24.6 ± 1.9	22.1 ± 2.2	23.2 ± 5.0	19.3 ± 3.4
***Chemotherapy regime***						
Administration route, *n* (%)						
*Metronomic (oral)*	19 (53)	5 (31)	5 (71)	6 (100)	-	3 (100)
*Standard (intravenous)*	17 (47)	11 (69)	2 (29)	-	4 (100)	-
Drug target, *n* (%)						
*Anti-mitotic (alkylating agents, vinca alkaloids)*	19 (53)	11 (69)	5 (72)	-	-	3 (100)
*Anti-DNA (alkylating agents, anti-metabolites)*	12 (33)	5 (31)	1 (14)	6 (100)	-	-
*Anti-mitotic/Anti-DNA (taxane/anthracycline, alkylating agent/taxane, alkylating agent/anti-microtubules agent)*	5 (14)	-	1 (14)	-	4 (100)	-

^a^PS: performance status according the ECOG/WHO scale; ^b^WBCs: white blood cells; ^c^SE: standard error.

### Purification of CD4^+^CD25^-^ and CD4^+^CD25^+^ T-cells

**Figure 1:**
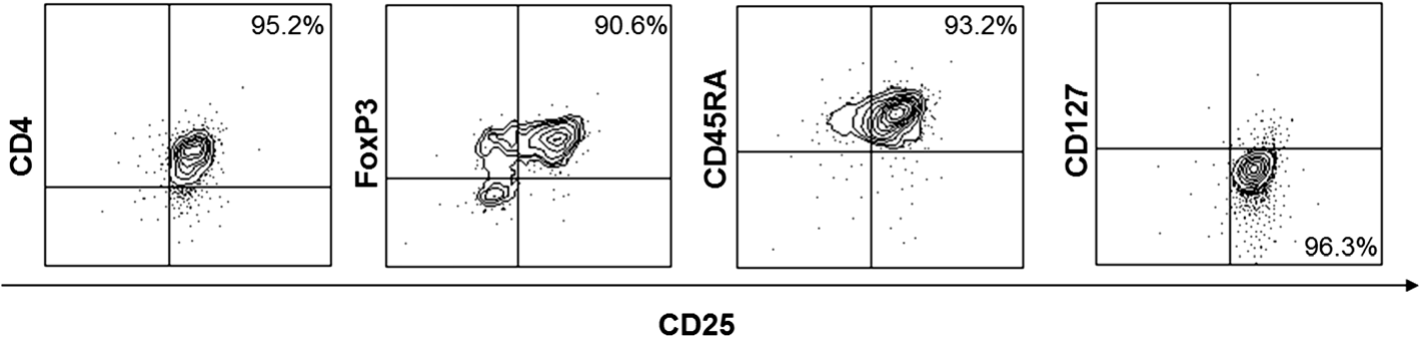
**Treg purity as assessed by flow cytometry.** Magnetically purified Tregs were stained with mAbs to human CD25, CD4, FOXP3 and CD45RA or CD25 and CD127. Numbers indicate percentages of positive cells in the purified population. Shown dot plots are from one representative cancer patient of 6 tested.

Peripheral blood mononuclear cells (PBMCs) were freshly isolated using density-gradient centrifugation on the appropriate polysaccharide (Histopaque-1077, Sigma Aldrich, Chemie GmbH, Germany). The CD4^+^CD25^-^ Teffs and CD4^+^CD25^+^ Tregs were separated by magnetic-bead sorting using the CD4^+^CD25^+^ regulatory T-cell isolation kit (Miltenyi Biotec, Bergisch Gladbach, Germany) according to the manufacturer’s instructions and as previously described [17,18]. Specifically, the isolation of Teffs and Tregs was performed in a two-step magnetic labelling procedure. Briefly, in the first step, isolated PBMCs were incubated with a cocktail of primary biotin-conjugated monoclonal antibodies (mAbs) against CD8, CD14, CD15, CD16, CD19, CD36, CD56, CD123, TCRγ/δ, and CD235a, and secondary anti-biotin mAbs (isotype mouse IgG) conjugated to microbeads. CD4^+^ T-cells were negatively selected by separation over an appropriate magnetic column (MACS® Separation LS Columns, Miltenyi Biotec) placed in a suitable magnetic field (MACS® Separator, Miltenyi Biotec). In the second step, the CD4^+^ T-cell fraction was incubated with CD25-conjugated microbeads. Positive and negative selection of labeled CD4^+^CD25^+^ Tregs and unlabeled CD4^+^CD25^-^ Teffs, respectively, was performed over a second column (MACS® Separation MS Columns). To further enrich the Treg fraction, CD4^+^CD25^+^ cells were additionally separated over a third column. Cell purity was assessed by flow cytometry. Cells were stained with APC-, FITC-, PE-, or PerCP/Cy5.5-conjugated mAbs to human CD25 (M-A251), CD4 (SK3), FOXP3 (259D/C7) and CD45RA (HI100), respectively, or CD25-APC and CD127-PE (A019D5) (all from BioLegend, San Diego, CA), according to manufacturer’s instructions. Samples were analysed using FACSCanto II (Becton-Dickinson (BD) Biosciences, Erembodegem, Belgium), while data acquisition and analysis were performed using the FACSDiva software (BD Biosciences) (Figure 1).

Treg and Teff frequency was expressed as percentage on gated CD4^+^ T-cells. The cell number ratio of Tregs to Teffs was exported from the formula: [%Tregs/CD4^+^ T-cells] / [%Teffs/CD4^+^ T-cells].

### CD4^+^CD25^-^ and CD4^+^CD25^+^ T-cell cultures

The suppressive capacity of CD4^+^CD25^+^ Tregs over CD4^+^CD25^-^ Teffs was examined in co-cultures of the two freshly purified T-cell subpopulations (Teffs + Tregs co-cultures) in the presence of CD2, CD3 and CD28 antibodies (Tregs Suppression Inspector; Miltenyi Biotec) following the manufacturer’s instructions and as described [17-19]. Suppression was initially estimated in selected samples using serial dilutions ranging from a Teff:Treg ratio of 1:1 to 8:1 and the ratio of 4:1 was selected for further screening. Control Teffs and Tregs were separately cultured with and without stimulus. All cultures were performed in triplicates (Additional file 1: Figure S1).

Briefly, Tregs and Teffs were suspended in RPMI 1640 (Biochrom AG, Berlin, Germany) supplemented with 10% heat-inactivated normal non-immune foetal bovine serum, 2 mM L-glutamine, 10 mM Hepes, 5 μg/mL gentamycin, 10 U/mL penicillin and 10 U/mL streptomycin (all from Gibco Life Technologies, GmbH, Germany) at 5×10^5^ cells/mL. Cells were distributed in round-bottom 96-well microtitre plates and incubated at 5% CO_2_ in a humidified chamber maintained at 37°C, for 96 hours.

### Proliferation assay

Cell proliferation of individually cultured and co-cultured CD4^+^CD25^-^ Teffs and CD4^+^CD25^+^ Tregs was evaluated using a colorimetric enzyme-linked immunosorbent assay (ELISA) kit (Cell Proliferation ELISA, BrdU-colorimetric, Roche Applied Science, Mannheim, Germany), according to the manufacturer’s instructions and as previously described [20]. Incorporation of the 5-bromo-2′-deoxyrinide (BrdU) pyrimidine analogue in the DNA of replicating cells, was determined as optical density (OD) measured at 450 nm with an ELISA reader (EL×800 Absorbance Microplate Reader, BioTek Instruments Inc, Bad Friedrichshall, Germany). The percentage of Teff suppression was calculated according to the formula: 1-(OD Teffs + Tregs / OD Teffs) × 100.

### Measurement of cytokine levels in culture supernatants

TGF-β and IL-10 levels in culture supernatants were evaluated with commercial ELISA kits (Quantikine Immunoassay, R&D System, MN, Minnesota, USA), according to manufacturer’s instructions. OD was measured at 450 nm using an ELISA reader (BioTek). All samples were tested in triplicates. Cytokine levels are expressed as concentration values in pg/mL.

### Statistical analysis

Statistical analysis of the data was performed with Mann–Whitney or paired-t tests, where appropriate, on the GraphPad-Prism 5.0 software (GraphPad Software, San Diego, CA). The tests were two-sided and the significance was defined as *p*-value < 0.05. Only statistically significant differences are reported.

## Results

### Metronomic chemotherapy increased the Tregs/Teffs ratio in the peripheral blood of cancer patients

**Figure 2:**
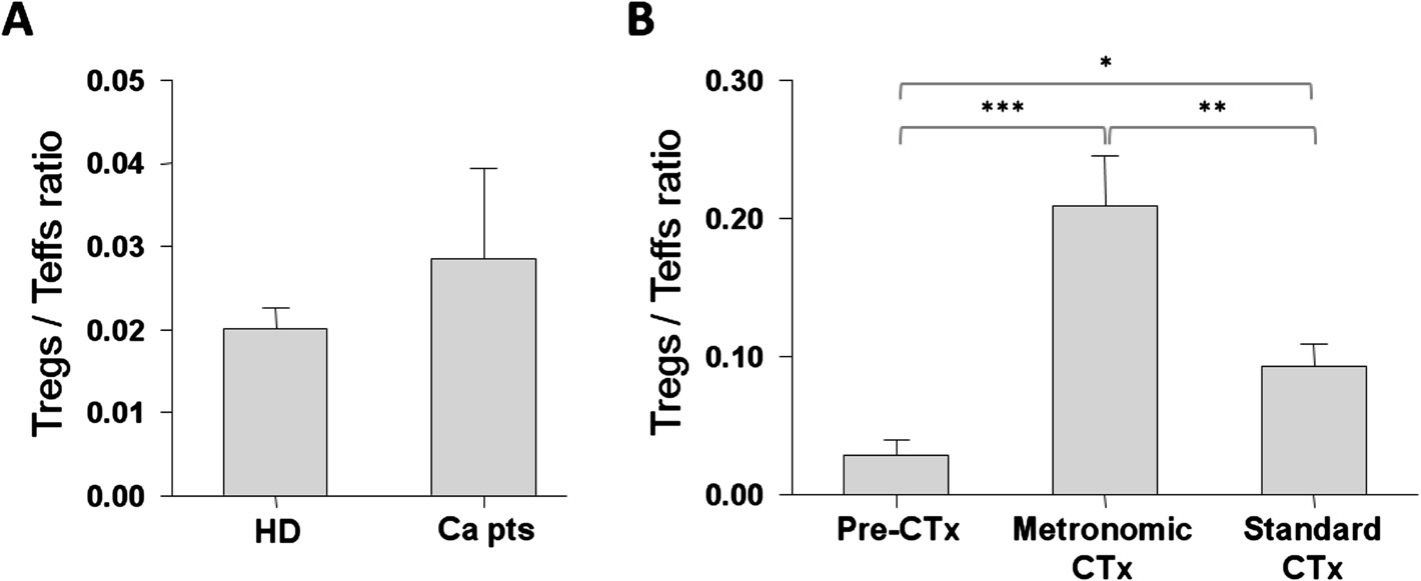
**Chemotherapy increases the number of Tregs.** Differential distribution of Treg/Teff ratio in the peripheral blood of **(A)** healthy donors (HD) and non-treated cancer patients (Ca pts), and **(B)** Ca pts before chemotherapy (Pre-CTx) and patients after the first cycle of metronomic or standard chemotherapy administration. All samples were analyzed in duplicates. Shown are mean ratios ± SE. *p < 0.05, ** *p* < 0.01; *** *p* < 0.001, as estimated by the Mann–Whitney test.

In accordance with previous reports [7], the Treg/Teff ratio was elevated in peripheral blood samples of non-treated cancer patients (mean ratio 0.029) in comparison to healthy donors (0.020; Figure 2A). After the first cycle of chemotherapy (metronomic or standard), these patients exhibited a statistically significant increase in peripheral Treg/Teff ratios (Figure 2B). It should be noted that this increase was more pronounced in patients treated with metronomic chemotherapy (0.209; *p* = 0.0007), than in those treated with standard chemotherapy (0.093; *p* = 0.021; Figure 2B). The differences recorded between the two chemotherapy groups were also statistically significant (*p* = 0.003; Figure 2B).

### The suppressive capacity of Tregs was more pronounced in metronomic than in standard chemotherapy-treated patients

**Figure 3:**
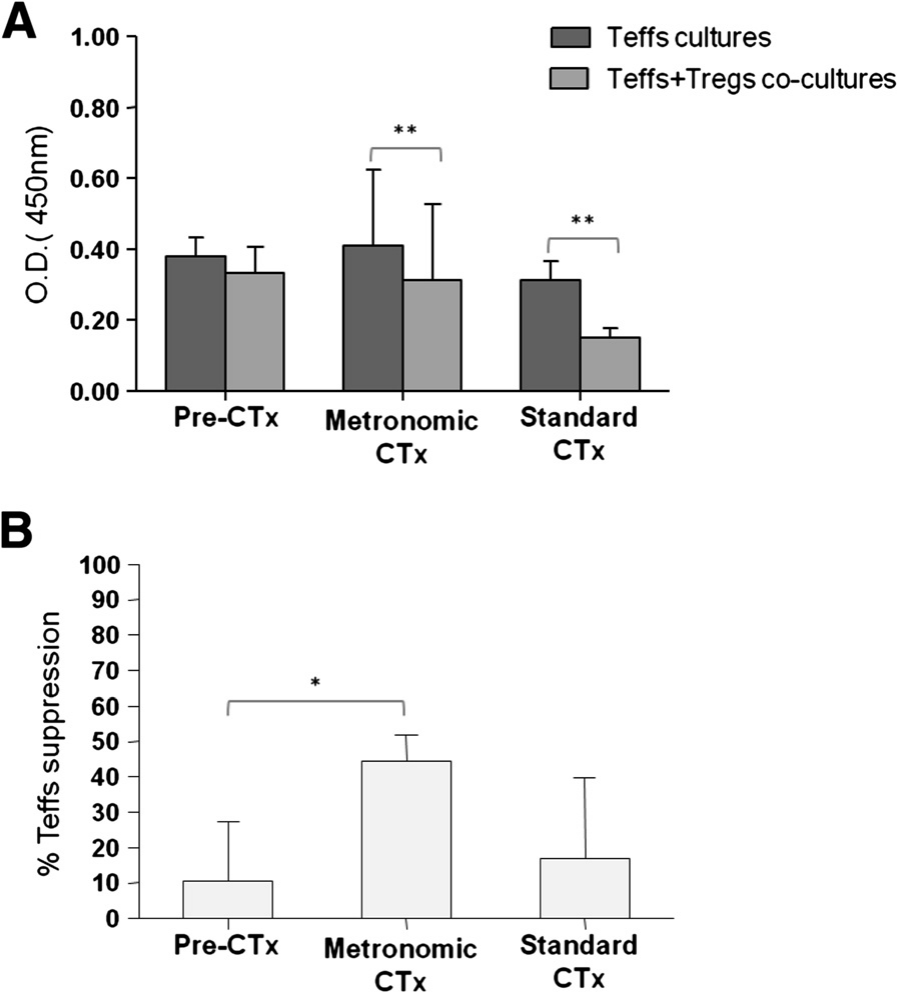
**Metronomic chemotherapy affects the number and function of Tregs. (A)** Teff proliferation was measured by the cell proliferation assay and expressed as mean optical densities (OD) at 450 nm ± SE. Teffs, single cultures of Teffs; Teffs + Tregs, co-cultures of Teffs with autologous Tregs at a ratio 4:1. **(B)** Treg-induced suppression of Teff proliferation. Shown are mean % values ± SE. Pre-CTx, patients before chemotherapy administration. All samples were analyzed in triplicates. * *p* < 0.05, ** *p* < 0.01, as estimated by paired-t test.

In accordance with previous data [17-19], the isolated peripheral blood CD4^+^CD25^-^ Teffs stimulated with CD2, CD3 and CD28 antibodies exhibited a marked proliferative response, while the stimulated CD4^+^CD25^+^ Tregs a hypo-proliferative response (anergy; Additional file 1: Figure S1). Moreover, the co-culture of stimulated Tregs and Teffs (Teffs + Tregs co-cultures) resulted in reduced proliferation of the latter (Figure 3A).

Of note, this reduction was greater after chemotherapy administration (metronomic or standard). Specifically, in patients who completed the first chemotherapy cycle, the proliferation rate of peripheral blood Teffs was significantly reduced when these cells were co-cultured with autologous Tregs (Teffs + Tregs co-cultures), compared to Teffs cultured alone (absolute OD 0.31 *vs* 0.41, for the metronomic treatment (*p* = 0.0016) and 0.15 *vs.* 0.31, for the standard treatment (*p* = 0.0099); for Teffs + Tregs *vs.* Teffs, respectively; Figure 3A). Although some reduction in Teff proliferation was also observed in samples from cancer patients prior to chemotherapy administration (0.33 *vs*. 0.38, for Teffs + Tregs *vs.*Teffs; Figure 3A), this was marginal compared to that noted in chemotherapy-treated patients.

More interestingly, by determining the percentage of Treg-induced suppression on Teff proliferation, we observed that this was much higher (by ~2.5-fold) in the peripheral blood of patients undergoing metronomic chemotherapy (44.42%), compared to suppression induced in patients treated with standard chemotherapy approaches (16.86%; Figure 3B). The lowest Treg-induced suppression was detected in untreated patients (10.65%). In accordance to this observation was the statistically significant difference in % Teff suppression recorded between the pre-treatment and metronomic treatment groups (*p* = 0.044, Figure 3B).

### Treg-induced suppression was associated with the type of chemotherapeutic drug administered

**Figure 4:**
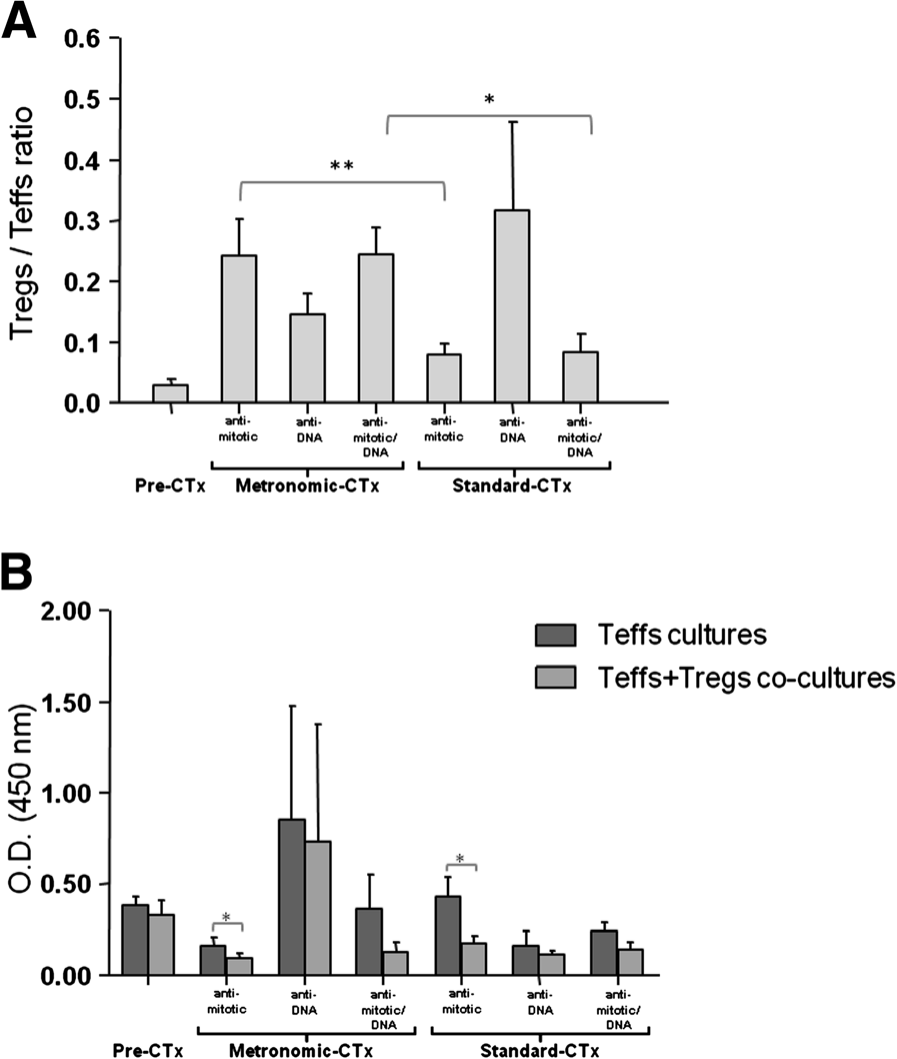
**Effect of the type and means of chemotherapy administration on Tregs. (A)** Differential distribution of Treg/Teff ratio in the peripheral blood of patients treated with anti-mitotic, anti-DNA or combined (anti-mitotic/anti-DNA) regimens in a metronomic or standard pattern. Shown are means ± SE. All samples were analyzed in duplicates. **(B)** Teff proliferation expressed as mean OD at 450 nm ± SE. All samples were analyzed in triplicates. Other details as in Figure 2. * *p* < 0.05, ** *p* < 0.01, as estimated by Mann–Whitney or paired-t test.

To assess whether the differential mode of action of chemotherapeutics impacts both on Treg number and functionality, we further classified chemotherapy-treated patients in subgroups based on the drug target: anti-mitotic (e.g. alkylating agents, vinca alkaloids, taxanes, anti-microtubules agents) and/or anti-DNA (e.g. alkylating agents, anti-metabolies, anthracyclines). Our results revealed that among the regimens applied in a metronomic pattern, administration of anti-mitotic and anti-mitotic/anti-DNA schemes was associated with higher Treg/Teff ratios (0.243 and 0.245, respectively), compared to anti-DNA drugs (0.146; Figure 4A). Moreover, the activity of Tregs from these 2 groups was increased, and co-culture of the two subpopulations, induced a significant suppression of autologous Teff proliferation (for the anti-mitotic drugs, 0.09 *vs.* 0.16 for Teffs + Tregs *vs.* Teffs, respectively; *p* = 0.018; for the anti-mitotic/anti-DNA combination, 0.36 *vs.* 0.13 for Teffs + Tregs *vs.* Teffs, respectively; Figure 4B). To reveal a potential mechanism of the recorded Teff suppression, we used cytokine-specific ELISAs to determine the levels of TGF-β and IL-10 in culture supernatants. Among the metronomically administrated drugs, the anti-mitotic group was associated with the highest levels of IL-10 (>2-fold) and TGF-β (>1.5-fold), compared to the anti-DNA group or the anti-mitotic/anti-DNA combination. These data suggest that the high numbers of Tregs detected in the peripheral blood of patients treated with metronomic chemotherapy, were active and secreted suppressive cytokines.

Within the group of patients receiving standard chemotherapy, the anti-DNA-treated subgroup showed the highest ratio of Tregs/Teffs (0.316 compared to 0.080 and 0.084 of the anti-mitotic- and anti-mitotic/anti-DNA-administered subgroups, respectively; Figure 4A). However, significant repression of Teff proliferation by Tregs was detected only in the anti-mitotic subgroup (0.09 *vs.* 0.16, for Teffs + Tregs *vs.* Teffs, respectively; *p* = 0.018; Figure 4B). This correlated with higher levels of suppressive cytokines characterizing the anti-mitotic subgroup (>2.5-fold for IL-10, and >2-fold for TGF-β), compared to the values determined in samples belonging to the anti-DNA or anti-mitotic/anti-DNA chemotherapy subgroups.

Further analysis of our results showed that metronomic administration of anti-mitotic agents, either alone or in combination with anti-DNA drugs, resulted in a higher Treg/Teff ratio, compared to standard administration (0.243 *vs.* 0.080, *p* = 0.0064 for the anti-mitotic agents; 0.245 *vs.* 0.084, *p* = 0.034, for the combination of anti-mitotic/anti-DNA regimens; Figure 4A).

The above referred observations suggest that anti-mitotic regimens, particularly if given metronomically, act in favour of Tregs more potently than anti-DNA agents or the combination of the two.

### Breast cancer patients treated with metronomic chemotherapy have increased numbers of functionally competent Tregs in their peripheral blood

**Figure 5:**
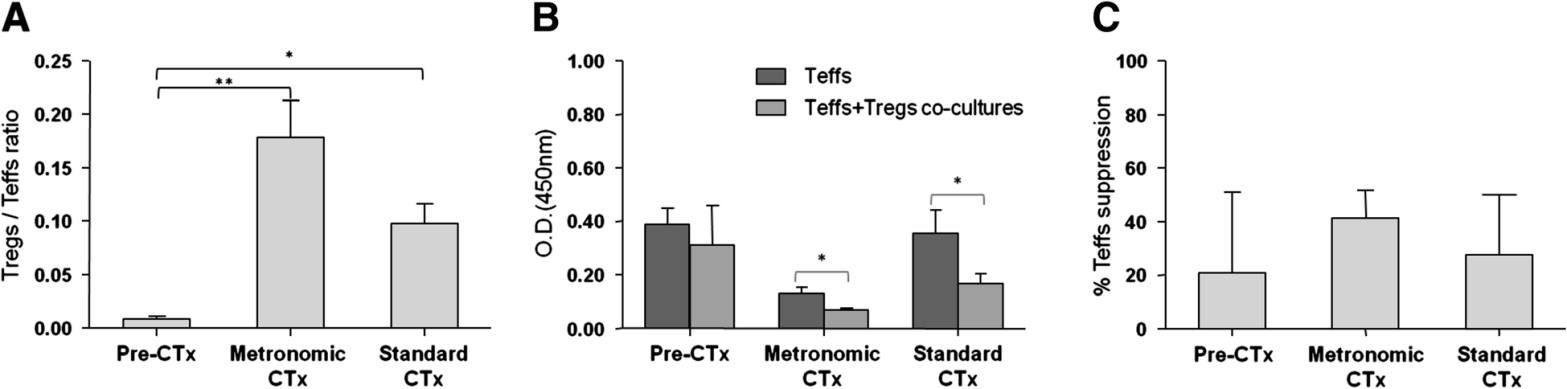
**Effect of metronomic and standard chemotherapy on breast cancer patients. (A)** Differential distribution of Treg/Teff ratio in the peripheral blood of breast cancer patients prior chemotherapy administration (Pre-CTx) and after metronomic or standard treatment. Shown are mean values ± SE. All samples were analyzed in duplicates. **(B)** Teff proliferation expressed as mean OD at 450 nm ± SE. **(C)** Treg-induced suppression on Teff proliferation. Shown are mean % values ± SE. All samples were analyzed in triplicates. Other details as in Figure 2. * *p* < 0.05 and ** *p* < 0.01 as estimated by Mann–Whitney or paired-t tests.

To further associate the effect of metronomic *vs* standard chemotherapy in a specific type of cancer, we selected breast cancer patients from our cohort and analysed both the number and the activity of peripheral blood Tregs. As shown in Figure 5, the favourable effect of chemotherapy on Treg/Teff ratio was more prominent in patients receiving metronomic than standard chemotherapy (0.179 and 0.098, respectively) and these values were much higher in both treatments compared to the Treg/Teff ratio in pre-chemotherapy breast cancer patients (0.009) (*p* = 0.0096 or *p* = 0.031 for pre-chemotherapy *vs.* metronomic or standard groups, respectively, Figure 5A). Accordingly, a statistically significant decrease in Teff proliferation upon co-culture with autologous Tregs was associated with the administration of either metronomic (0.13 *vs.* 0.69 for Teffs *vs.* Teffs + Tregs, respectively; *p* = 0.04) or standard treatment (0.36 *vs.* 0.17 for Teffs *vs.* Teffs + Tregs, respectively; *p* = 0.05) (Figure 5B). However, higher percentages of Tregs-induced suppression of Teffs (41.10%) were noticed in the group of patients treated with metronomic chemotherapy compared to that estimated after standard administration of the agents (27.59%), as well as in pre-chemotherapy breast cancer patients (20.77%; Figure 5C). These results, although preliminary and acquired using a limited number of samples, show that even one cycle of metronomic chemotherapy administration increases the suppressive functions of Tregs, and suggest that prolonged treatment may possibly abrogate the integrity of immune responses.

## Discussion

For over a decade, metronomic chemotherapy has been proposed as an alternative to conventional drug administration in cancer patients. Metronomic administration comprises 3 characteristics: (1) it is frequent, often on a daily basis; (2) it is continuous, with no drug-free breaks and (3) the doses are lower than the maximum tolerated dose [21,22]. Its low toxicity and cost, ease of administration and efficacy justify the continuously increasing number of reports, both in animals and in humans, in support of its use. Metronomic chemotherapy, although initially shown to inhibit angiogenesis and reduce the levels of some biological markers (e.g. VEGF) is now proven to modulate immune responses, by increasing the *de novo* generation of tumor-specific effector T-cells, re-sensitize pre-existing suppressed tumor-reactive T-, NK- and NKT-mediated responses and induce the maturation of dendritic cells [21,23]. Numerous studies addressed the effect of metronomic chemotherapy on specific types of cancer, using a specific drug and a specific regime [22,24]. However, only few reports refer to the direct comparison of immune parameters in metronomic *vs*. standard chemotherapy in cancer patients [22,25]. This prompted us to study the possible derangement, both in cell numbers and functionality, of the peripheral effector/regulatory T-cell equilibrium in patients with various solid tumors, using chemotherapeutics with different, albeit precise, modes of action, focusing in principle on the comparative investigation between metronomic and standard chemotherapy strategies. In contrast with earlier reports, where either the Tregs or Teffs were examined, this study explores the chemotherapy impact on the balance between the two, regulatory and effector, T-cell subpopulations. Moreover, the functional and numerical parameters of Tregs/Teffs are investigated in association with the manner of chemotherapy administration (metronomic or standard) and the mode of action of the chemotherapeutic agent (anti-mitotic or anti-DNA or both).

Due to several inconsistencies as for the identification of peripheral blood Tregs and in order to ensure that the populations studied herein are those of interest, we initially evaluated our T-cell (Tregs and Teffs) purification protocol in healthy donors and cancer patients prior and during chemotherapy. In agreement with data available in the literature [26-29], healthy donors had low numbers of Tregs, which increased in cancer patients and were further augmented upon chemotherapy administration. Moreover, irrespective of the means of administration (metronomic or standard), chemotherapy reduced the relative numbers of CD4^+^CD25^-^ Teffs and augmented the suppressive effect of Tregs on Teffs. Our results are in line with previous reports where patients with haematologic malignancies demonstrated a marked Teff suppression, indicating that lymphocyte recovery after chemotherapy may augment Treg proliferation [20]. Lymphopenia characterizing cancer patients offers a possible explanation to the observed Treg enforcement, as described by Haribhai *et al.* (2009) [30]. In a lymphopenic environment, members of the naïve T-cell repertoire, irrespective of their specificity, may be remodelled, join the suppressive T-cell compartment and finally contribute to the deregulation of Teff expansion [30].

In our cohort of patients, metronomic administration exerted a more intense immunosuppressive effect compared to standard treatment, acting in favour of Tregs and against Teffs. As shown, metronomic chemotherapy increased the number of Tregs and the secretion of TGF-β and IL-10, and reduced Teff proliferation. The increase in the number of Tregs we observed, is in contrast to most studies published to date which suggest that metronomic approaches deplete Tregs and improve cancer patients’ immunocompetence [22,25]. However, our data are in agreement with the most recent elegant study of Ge *et al.*[31] revealing that metronomic cyclophosphamide treatment only transiently reduced the number of Tregs in breast cancer patients and Treg proliferation fully recovered after 4–6 weeks of ongoing treatment, and with Ellebaek *et al.*[32] who showed a pronounced Treg increase in melanoma patients treated with metronomic cyclophosphamide administered in conjunction with a Cox-2-inhibitor and a DC-based multi-epitope vaccine. The reasons for the contradictory effects of metronomic chemotherapy on Tregs are not clear. They could be explained by the significantly higher regeneration rate of Tregs (approximately 8 days) compared to Teffs (24 days for memory; 199 days for naive) [33], allowing Tregs to return to their normal cell cycling and growth in the thymus or in peripheral lymph nodes, and their prompt re-enter in circulation. Teffs cannot be as rapidly recruited, since following their activation, antigen-stimulated T-cells have been reportedly shown to enter a transient refractory state that lasts for several additional days [17]. We speculate that the doses and the schedule of drug administration, the type and stage of cancer, the mode of action of the chemotherapeutic drug, the basal status of the immune system of each patient at treatment initiation, or combinations thereof may additionally account for the inconsistencies reported. For example, the Treg/Teff balance may be relevant to the divergent bioavailability of orally (metronomic) and intravenously (standard) administered drugs, associated with different opportunities for the Treg and Teff populations to recover. Moreover, in regard to the specific drugs mode of action, and although our analysis was performed on a small number of samples, our data indicate that chemotherapeutics targeting the microtubules of the spindle during mitosis (anti-mitotic drugs) are associated with higher Treg numbers and an increased Treg-induced suppression of Teffs, in comparison with those regimens that interfere with the DNA sequence during the S phase of the cell-cycle (anti-DNA drugs), or a combination of the two. Although ongoing *in vitro* experiments will explicate our initial observation, these variations in the drugs’ targets could be associated with the aforementioned differences in cell cycling between Tregs and Teffs.

The functional analysis of Tregs expanded *in vivo* upon metronomic chemotherapy verified their ability to suppress specific immune activities. When Tregs purified from the peripheral blood of patients treated with one cycle of metronomic chemotherapy were cultured *ex vivo*, they secreted high levels of IL-10 and TGF-β and significantly suppressed autologous Teff proliferation. Teff suppression was more prominent in patients receiving anti-mitotic drugs and was positively correlated both with the recorded high Treg/Teff ratios and the determined increased levels of IL-10 and TGF-β.

No matter the reason(s), the finding that metronomic administration enhances the number and function of Tregs, as shown by us (this report) and others [31,32], is essential and should be considered when selecting the appropriate chemotherapy strategy, particularly in cases where the use of chemotherapy prior to vaccination may hinder the efficacy of the vaccine. Since combined vaccination and chemotherapy strategies often fail to show survival benefit, this could merely be an underlying cause [16].

Due to its advantages, the application of chemotherapy in a metronomic manner is a desirable objective, especially in types like breast cancer, where the patient can be treated at home, experience less toxic side effects and avoid hospitalization [21,22]. However, the selective analysis of our breast-cancer group indicated that metronomic chemotherapy enforced the Treg population to a greater extent than standard administration. Hence, confirming our results from the entire cohort, these data suggest that metronomic compared to standard chemotherapy, results in a more unfavourable, as for the patients’ outcome, Treg-Teff interaction.

We cannot exclude the possibility that the shift in Treg/Teff ratio observed in this study may reflect more complex underlying mechanisms among the host, the tumour and the regimen. Although the Treg-Teff tug-of-war is important, other immune components that may also be affected by chemotherapy play a crucial role in the host’s homeostasis, including myeloid-derived suppressors [5,6], macrophages and/or NK cells [34-39]. Thus, the contribution of the reported chemotherapy-enhanced Treg suppressive activity against Teffs to tumour immune-escape, needs to be further explored.

More studies are warranted to clarify the impact of the interplay between chemotherapeutic agents and immune cells in the effectiveness of the anti-tumor immunity and, therefore, in the success of the treatment strategy. The extensive exploration of each tumour’s features in association with the immunological profile of the patient is vital for the development of personalised therapeutic interventions, where the manner of administration and the specific mode of action of the regimen should be also taken into account.

## Conclusion

The current study supports that in comparison with standard anti-cancer treatment strategies, the alternative approach of metronomic chemotherapy, though more patient-friendly, prominently acts in favour of Tregs, and impairs the regulatory-to-effector T-cell imbalance against the host’s anti-tumor immunity. The findings on this immune-related impact of chemotherapy may be proven useful in the clinicians’ selection of the most advantageous drug-delivery strategy, particularly in cases when immunotherapeutics are eventually to be applied.

## Abbreviations

CTx: Chemotherapy; p.o.: Oral administration; i.v.: Intravenous administration; Tregs: Regulatory T lymphocytes; Teffs: Effector T lymphocytes; PBMCs: Peripheral blood mononuclear cells; vs.: *versus*; SE: Standard error.

## Competing interests

The authors declare that they have no competing interests.

## Authors’ contributions

AK conceived the idea of the study, designed and supervised the project. She had also a principal contribution in material support, acquisition, analysis and interpretation of data as well as in writing the manuscript. MIC had a major contribution in the acquisition, analysis and interpretation of data, as well as in writing the manuscript. PP, NP, NX, NT, and DP were involved in material support and revision of the manuscript. IP, AG, and AS contributed in data acquisition and revision of the manuscript. EL contributed in the development of methodology and revision of the manuscript. TE was involved in analysis and interpretation of data and revision of the manuscript. OT had a principal contribution in analysis and interpretation of data as well as in writing and reviewing the manuscript. VP was involved in the development of methodology, analysis and interpretation of data and revision of the manuscript. All authors read and approved the final manuscript.

## Supplementary Material

Additional file 1: Figure S1 Titration of Teff:Treg ratio in co-cultures. Serial dilutions of Teffs:Tregs were tested in a range 8:1 to 1:1. Control Teffs and Tregs were separately cultured with and without stimulus. All cultures were performed in triplicates. Shown data are from 3 cancer patients of 36 tested in total. Click here for file
