# Supplementary figures and images for: The effect of metronomic versus standard chemotherapy on the regulatory to effector T-cell equilibrium in cancer patients
Source: Exp Hematol Oncol. 2014 Jan 23;3:3. doi: 10.1186/2162-3619-3-3 (PMC3906764; doi:10.1186/2162-3619-3-3)

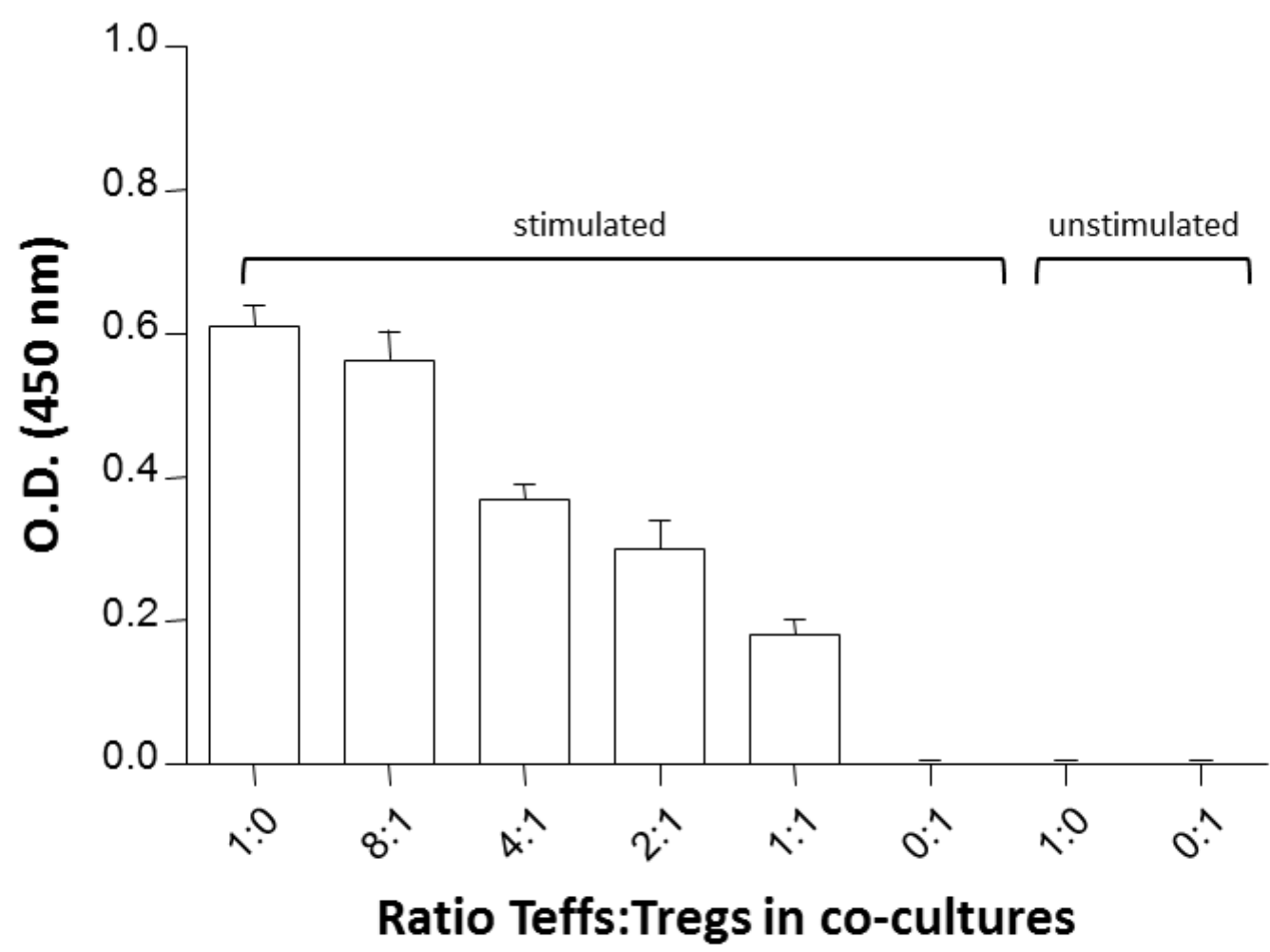

Supplement: Additional file 1: Figure S1 — Titration of Teff:Treg ratio in co-cultures. Serial dilutions of Teffs:Tregs were tested in a range 8:1 to 1:1. Control Teffs and Tregs were separately cultured with and without stimulus. All cultures were performed in triplicates. Shown data are from 3 cancer patients of 36 tested in total. [file 2162-3619-3-3-S1.pdf]
